# Identification of Novel Independent Correlations between Cellular Components of the Immune System and Strain-Related Indices of Myocardial Dysfunction in CKD Patients and Kidney Transplant Recipients without Established Cardiovascular Disease

**DOI:** 10.3390/ijms25179162

**Published:** 2024-08-23

**Authors:** Anila Duni, Athanasios Kitsos, Aris Bechlioulis, Lampros Lakkas, Georgios Markopoulos, Vasileios Tatsis, Vasileios Koutlas, Eirini Tzalavra, Gerasimos Baxevanos, Georgios Vartholomatos, Michail Mitsis, Katerina K. Naka, Evangelia Dounousi

**Affiliations:** 1 Department of Nephrology, Faculty of Medicine, School of Health Sciences, University Hospital of Ioannina, University of Ioannina, 455 00 Ioannina, Greece; anikristduni@yahoo.com (A.D.); thkitsos@hotmail.com (A.K.); 2 Kidney Transplant Unit, Department of Surgery, Faculty of Medicine, School of Health Sciences, University Hospital of Ioannina, University of Ioannina, 455 00 Ioannina, Greece; tatsis.vasileios@hotmail.com (V.T.); v_koutlas@yahoo.gr (V.K.); tzalavrairini@yahoo.gr (E.T.); mmitsis@uoi.gr (M.M.); 3 Second Department of Cardiology, Faculty of Medicine, School of Health Sciences, University Hospital of Ioannina, University of Ioannina, 455 00 Ioannina, Greece; md02798@yahoo.gr (A.B.); ftpcavalier52@gmail.com (L.L.); drkknaka@gmail.com (K.K.N.); 4 Laboratory of Haematology—Unit of Molecular Biology and Translational Flow Cytometry, University Hospital of Ioannina, 455 00 Ioannina, Greece; geomarkop@gmail.com (G.M.); gbaxevanos@gmail.com (G.B.); gvarthol@gmail.com (G.V.); 5 Department of Internal Medicine, General Hospital of Ioannina, G. Chatzikosta, 454 45 Ioannina, Greece

**Keywords:** CKD, kidney transplantation, cardiac remodeling, subclinical myocardial dysfunction, left ventricular strain, classical CD14++CD16− monocytes, intermediate CD14++CD16+ monocytes, CD4+ T-cells, CD8+ T-cells, natural killer cells

## Abstract

The role of immune system components in the development of myocardial remodeling in chronic kidney disease (CKD) and kidney transplantation remains an open question. Our aim was to investigate the associations between immune cell subpopulations in the circulation of CKD patients and kidney transplant recipients (KTRs) with subclinical indices of myocardial performance. We enrolled 44 CKD patients and 38 KTRs without established cardiovascular disease. A selected panel of immune cells was measured by flow cytometry. Classical and novel strain-related indices of ventricular function were measured by speckle-tracking echocardiography at baseline and following dipyridamole infusion. In CKD patients, the left ventricular (LV) relative wall thickness correlated with the CD14++CD16− monocytes (β = 0.447, *p* = 0.004), while the CD14++CD16+ monocytes were independent correlates of the global radial strain (β = 0.351, *p* = 0.04). In KTRs, dipyridamole induced changes in global longitudinal strain correlated with CD14++CD16+ monocytes (β = 0.423, *p* = 0.009) and CD4+ T-cells (β = 0.403, *p* = 0.01). LV twist and untwist were independently correlated with the CD8+ T-cells (β = 0.405, *p* = 0.02 and β = −0.367, *p* = 0.03, respectively) in CKD patients, whereas the CD14++CD16+ monocytes were independent correlates of LV twist and untwist in KTRs (β = 0.405, *p* = 0.02 and β = −0.367, *p* = 0.03, respectively). Immune cell subsets independently correlate with left ventricular strain and torsion-related indices in CKD patients and KTRs without established CVD.

## 1. Introduction

Cardiac remodeling is a hallmark of chronic kidney disease (CKD) manifesting as myocardial fibrosis, left ventricular hypertrophy (LVH), impaired myocardial strain and eventually left ventricular diastolic and systolic dysfunction [1,2]. Myocardial fibrosis is evident since the initial stages of CKD and augments with disease progression [3,4]. Likewise, cardiac imaging studies utilizing echocardiography or MRI have shown that LVH is detected early in CKD and affects approximately 40% of non-end-stage CKD patients and more than 75% of dialysis patients [5,6,7]. Myocardial deformation analysis data from speckle-tracking echocardiography (STE) studies have revealed subclinical abnormalities of the left ventricular myocardial relaxation and contractile function in CKD patients in the absence of established cardiovascular disease CVD [7,8]. Specifically, left ventricular global longitudinal strain (GLS) has emerged as a more sensitive predictor of mortality compared to ejection fraction (EF) in these patients [9].

Kidney transplantation is associated with significant improvements in left ventricular size and function as well as regression of LVH, otherwise known as reverse remodeling [10,11]. Nevertheless, subclinical abnormalities in the biventricular strain may be observed in kidney transplant recipients (KTRs) even when other classical indices of myocardial function such as EF are normal [12,13]. Remarkably, heart failure (HF) following kidney transplantation remains responsible for the majority of adverse cardiovascular events in KTRs [14,15].

The potential involvement of immune pathways in the pathogenesis of HF has come under the spotlight with the deleterious role of proinflammatory cytokines in the myocardium underlying the inflammatory paradigm of heart failure [16,17]. Markers of monocyte activation such as Toll-like receptor 4 (TLR4) and monocyte-derived cytokines, including TNF and IL-6, are increased in the peripheral blood and the myocardium in the setting of HF [18,19,20]. In vitro cultures of peripheral blood monocytes and human cardiac myofibroblasts indicate that monocytes increase myofibroblast activity through release of TGF-β1, leading to extracellular matrix deposition [21]. Monocytes, however, are a heterogenous population of three specific subsets, and their distinct roles in the pathogenesis of HF are currently under investigation. Accordingly, the classical CD14++CD16− monocytes are scavenger cells differentiating into macrophages in inflamed tissues, the intermediate CD16++CD14+ monocytes possess pro-oxidant, proinflammatory as well as proangiogenic properties and finally the least characterized CD14+CD16++ non-classical monocytes are considered responsible for clearing of vascular debris [22,23,24]. With regard to the acquired immune system cells, the contribution of T-lymphocytes to the development of LVH, fibrosis and subsequent progression to HF has recently gained increasing attention [25]. In the setting of pressure overload, activated T-cells display enhanced properties with respect to endothelial adhesion and left ventricular infiltration, which are to a certain degree mediated by IFN-γ [26].

The chronic inflammatory state and immune system activation are considered as significant mediators in the pathogenesis of myocardial dysfunction in CKD [27]. The role of monocyte subsets as well as T-lymphocytes and B-lymphocytes activation as putative factors causally implicated in myocardial dysfunction in the setting of CKD remains an open question. The intermediate CD14++CD16+ monocytes have been directly associated both with the atherosclerosis burden as well as with the occurrence of adverse atherosclerotic cardiovascular events in patients without and with CKD [28,29]. Recently, in a mouse model of uremic cardiomyopathy, T-cell depletion led to improved diastolic function as manifested by improved E/A ratio, isovolumic relaxation time, and myocardial performance index as well as improved myocardial strain [30]. Notably, the immune senescence of CKD is characterized among others by a major decrease in the naive T-cell populations and expansion of terminally differentiated CD4+ and CD8+ T-cells, which appear to be directly associated with increased cardiovascular risk in CKD patients and accelerated atherosclerosis in KTRs [31,32]. In a previous study, we demonstrated that patients with type 2 cardiorenal syndrome exhibit alterations in the immune cell profile compared to CKD patients of similar kidney function but without CVD [33]. We showed in the same cohort that CD4+ T-lymphocytes independently predicted mortality and fatal cardiovascular events [33]. Considering the promising evidence from clinical trials targeting inhibition of interleukin-1 and interleukin-6-mediated pathways in patients with atherosclerosis and CKD, the identification of specific immune phenotypes associated with myocardial dysfunction in the setting of CKD would be of major clinical significance [34].

There is scarce clinical evidence available regarding the implication of immune cell subsets in the development of CKD associated cardiomyopathy. We therefore conducted a pilot cross-sectional study in a cohort of non-end-stage CKD patients and kidney transplant recipients (KTRs) without established CVD to investigate for the first time the relation of a selected panel of immune cell subpopulations in the peripheral circulation with classical and novel, strain-related indices of myocardial performance as evaluated by two-dimensional STE.

## 2. Results

Table 1 summarizes the main baseline characteristics of the study cohort.

The mean age of patients with CKD was 63 ± 11 years, whereas KTRs had a mean age of 53 ± 9 years, respectively (*p* < 0.001), and most patients were males in both groups. Diabetics were 39% of CKD patients and 21% of KTRs while the majority of patients in both groups had arterial hypertension. Regarding kidney function markers, KTRs displayed significantly higher median eGFR compared to CKD patients [55 (IQR, 48–72) versus 24 (IQR, 15–41) mL/min/1.73 m^2^, respectively (*p* < 0.001)]. Likewise, the median UPCR was significantly lower in KTRs (0.16 g/g (IQR, (0.09–0.56)) versus 1.31 (IQR, (0.23–2.61)) g/g (*p*< 0.001) in CKD patients.

The vast majority of KTRs were on a triple immunosuppressive regimen including corticosteroids, a calcineurin inhibitor (CNI) and an antimetabolite (mycophenolate mofetil or mycophenolic acid). As for CNIs, 60% of KTRs were under treatment with tacrolimus, whereas 40% received cyclosporine. Finally, no significant differences were observed between the two groups regarding treatment with statins, angiotensin-converting enzyme inhibitors (ACEI) and angiotensin receptor blockers (ARB) or b-blockers.

### 2.1. Differences in the Profile of Immune Cell Subset Expression between CKD Patients and KTRs

**Figure 1:**
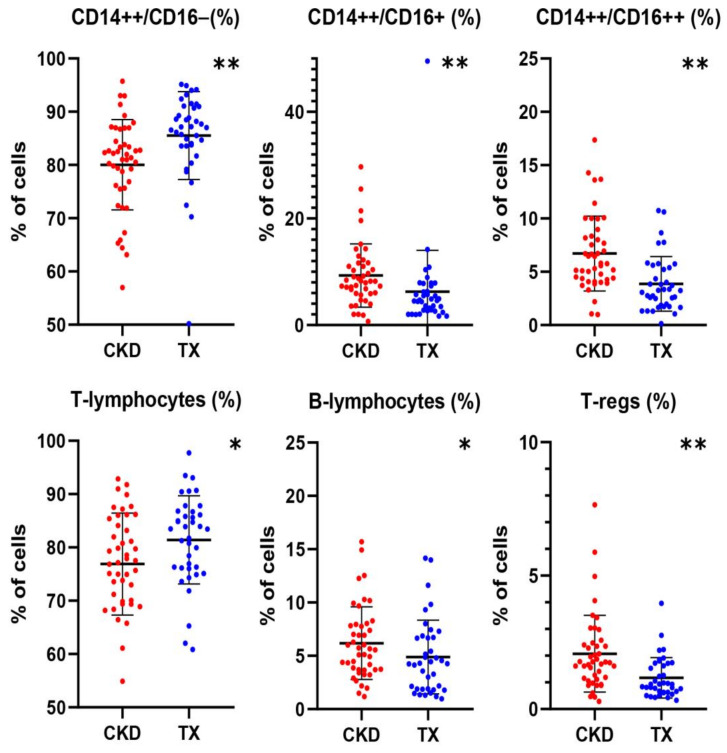
Immune cell subpopulations count in CKD patients and KTRs. * *p* < 0.05, ** *p* < 0.01. Values are expressed as the means or medians.

The differences between the peripheral blood levels of immune cell subpopulations between CKD patients and KTRs are shown in Table 2 and Figure 1. KTRs displayed both a higher number and percentage of classical CD14++CD16− monocytes [479 IQR (354–599)/μL and 87.1 (IQR, 83.6–90.1)%] compared to their CKD counterparts [366 (IQR, 258–438)/μL and 81.7 (IQR, 75.9–85.6)%, *p* = 0.001 and 0.000, respectively]. On the other hand, the percentage of intermediate CD14++CD16+ monocytes was lower in KTRs [4.6 (IQR, 2.8–7.3)%] compared to CKD patients [8.2 (IQR, 5.9–11.3)%, *p* < 0.001]. Similarly, the number and percentage of the non-classical CD14+CD16++ monocytes were lower in KTRs [18 (IQR 13–28)/μL and 3.2 (IQR, 1.9–5.4)%] compared to their CKD counterparts [25 (IQR, 19–36)/μL and 5.8 (IQR, 4.3–8.2)%, *p* = 0.001 and 0.000, respectively]. With regard to lymphocyte subpopulations, KTRs displayed a higher percentage of T-lymphocytes (81.4 ±8.3%) and a lower percentage of B-lymphocytes [(4.3 (IQR, 1.9–6.7)%] compared to CKD patients [76.7 ± 8.3%, *p* = 0.02 and 5.6 (IQR, 3.7–7.9)%, *p* = 0.04, respectively]. Finally, KTRs had lower number and percentage of Tregs [19 (IQR, 13–28)/μL and 0.93 (IQR, 0.63–1.71)%, respectively] compared to CKD patients [33 (IQR, 19–48)/μL and 1.75 (IQR, 1.13–2.44)%, *p* = 0.002 and *p*< 0.001, respectively]. Following adjustment for confounders including age, eGFR and UPCR, the differences in immune cell subsets between the two groups remained statistically significant for the percentage of classical monocytes (*p* =0.02, both the number and percentage of non-classical monocytes (*p* < 0.001), the percentage of T-lymphocytes and B lymphocytes (*p* = 0.03 and *p* = 0.003, respectively) as well as the Tregs number (*p* = 0.008).

### 2.2. Correlations of Immune Cell Subsets with Clinical and Laboratory Parameters in CKD Patients and KTRs

Univariate associations of various immune cell subtype levels with other clinical and laboratory parameters in patient subgroups are shown in Appendix A, Table A1 and Table A2. Regarding kidney function markers, eGFR (CKD-EPI) was directly associated with total lymphocytes count (r = 0.388, *p* = 0.009), T-cell counts (r = 0.328, *p* = 0.03) and CD4+ T-cells (r = 0.495, *p* = 0.001) in CKD patients (Appendix A, Table A1 and Table A2). In line with the above, a positive correlation was found between the eGFR, and total lymphocytes counts (r= 0.359, *p* = 0.027), T-cell counts (r = 0.376, *p* = 0.02) and CD8+ T-cell (r = 0.362, *p* = 0.02) in KTRs. Furthermore, UPCR was inversely correlated with the percentage of total lymphocytes (r = −0.439, *p* = 0.003) as well as with NK cell counts (r = −0.302, *p* = 0.04) in CKD patients and with B-lymphocytes counts (r = −0.405, *p* = 0.01) in KTRs, respectively (Appendix A, Table A1 and Table A2). Regarding CKD-MBD markers, serum phosphorus levels were directly correlated to intermediate CD14++CD16+ monocytes count both in CKD patients (r = 0.436, r = 0.003) and in KTRs (r = 0.333, *p* = 0.04 (Appendix A, Table A1 and Table A2).

### 2.3. Correlations of Immune Cells with Classical and Novel Indices of Left Ventricular Function in CKD Patients

**Figure 2:**
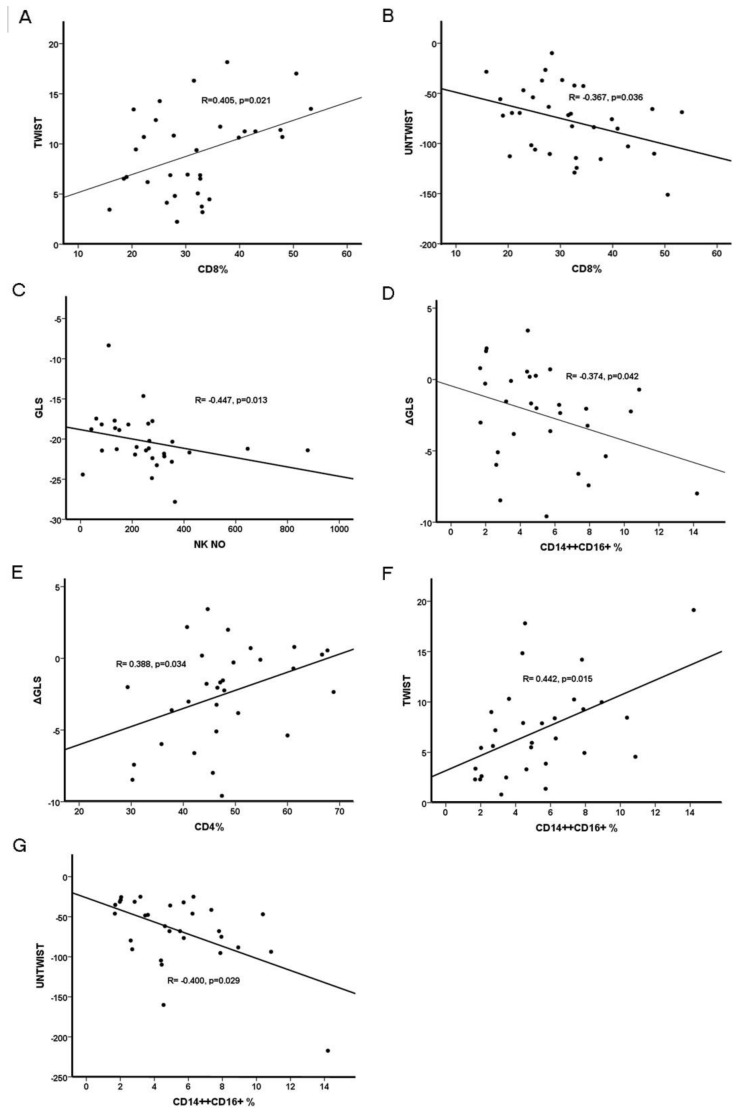
Associations between myocardial strain echocardiographic indices of cardiac function with immune cell subsets in patients with CKD and kidney transplant recipients. (**A**) Higher baseline left ventricular TWIST (better) is positively associated with the percentage of CD8+ T-cells in CKD patients. (**B**) More negative baseline left ventricular UNTWIST (better) is inversely associated with the percentage of CD8+ T-cells in CKD patients. (**C**) More negative (better) baseline GLS is inversely associated with NK cells in kidney transplant recipients. (**D**) DIPSE-induced improvement in GLS is associated with a higher percentage of CD14++CD16+ monocytes in kidney transplant recipients. (**E**) DIPSE-induced improvement in GLS is associated with a lower percentage of CD4+ T-cells in kidney transplant recipients. (**F**) Higher baseline left ventricular TWIST (better) is positively associated with the percentage of CD14++CD16+ monocytes in kidney transplant recipients. (**G**) More negative baseline left ventricular UNTWIST (better) is negatively associated with the percentage of CD14++CD16+ monocytes in kidney transplant recipients.

The final analysis of the echocardiographic data included 38 patients with CKD (6 patients were excluded due to poor acoustic window). The baseline values and post-dipyridamole changes in various echocardiographic parameters in CKD patients are shown in Appendix A, Table A3. Univariate and multivariate association analysis of classical and novel echocardiographic indices and their changes post-dipyridamole with immune cell subsets in CKD patients are shown in Table 3 and Figure 2.

Left ventricular relative wall thickness (RWT) was independently correlated with the classical CD14++CD16− monocytes count (β = 0.447, *p* = 0.004) and the percentage of B lymphocytes (β = −0.328, *p* = 0.03). Left ventricular mass index (LVMI) was found only to correlate with the percentage of total lymphocytes (r = −0.397, *p* = 0.02). Left ventricular ejection fraction (EF) was negatively associated with both the number and percentage of total lymphocytes (r = −0.345, *p* = 0.04 and r = −0.353 *p* = 0.03, respectively), the number of T-cells (r = −0.364, *p* = 0.023) as well as with the number of CD4+ lymphocytes (r = −0.451, *p* = 0.006), which remained an independent correlate of left ventricular EF (β = −0.431, *p* = 0.009).

No significant correlates were found for septal mitral annular systolic excursion (MAPSE) at baseline except for a borderline negative association with CD14++CD16− monocytes count (r = −0.320, *p* = 0.057), whereas the septal ΔMAPSE was independently correlated with the number of CD14++CD16+ monocytes (β = −0.359, *p* = 0.007). No other significant correlations were found between immune cell subsets and the rest of the measured classical echocardiographic indices.

Notably, left ventricular GLS at baseline was only associated with the ESR (r = −0.377, *p* = 0.026), but no associations were found between GLS and the ΔGLS with immune cell subpopulations. Significant correlates of the left ventricular global radial strain (GRS) included both the number and percentage of the CD14++CD16+ monocytes (r = 0.042, *p* = 0.01, and r = 0.352, *p* = 0.04). The association of ΔGRS with the CD14++CD16+ monocytes number (r = 0.331, *p* = 0.006) was lost at multivariate regression analysis. Left ventricular TWIST was independently associated with the percentage of CD8+ T-cells (β = 0.405, *p* = 0.02). Similarly, the CD8+ T-cells percentage was independently related to left ventricular UNTWIST (β = −0.363, *p* = 0.03).

### 2.4. Correlations of Immune Cells with Classical and Novel Indices of Left Ventricular Function in KTRs

Following exclusion of 7 KTRs due to poor acoustic window, the echocardiographic analysis included 31 KTRs. The baseline values and post-dipyridamole changes in various echocardiographic parameters in KTR patients are shown in Appendix A, Table A3. Univariate and multivariate association analysis of classic and novel echocardiographic indices and their changes post-dipyridamole with immune cell subtypes in KTR patients are shown in Table 4 and Figure 2.

Left ventricular EF was independently correlated with Tregs counts (β = −0.341, *p* = 0.03), whereas significant independent correlates of the ΔEF were the number of CD4+ T-cells (β = −0.378, *p* = 0.02). The tricuspid annular plane systolic excursion was associated with CD8+ T-cells (β = 0.559, *p* = 0.00). Septal MAPSE was independently correlated with CD4+ T-cell counts (β = −0.463, *p* = 0.04). Regarding the medial and lateral wall systolic velocity of the left ventricle (Sm and Sl, respectively), post-dipyridamole changes in Sm and Sl were independently associated with the percentage of CD14++ monocytes (β = −0.516, *p* = 0.01 and β = −0.707, *p* < 0.001, respectively). Finally, an independent correlation with NK cells number (β = −0.387, *p* = 0.02) was found for E/A ratio. All associations of E/E’ with immune cell subsets at univariate analysis, including the percentage of lymphocytes (r = −0.390, *p* = 0.03), CD4+ T-cells (r = −0.456, *p* = 0.01) as well as the Tregs number (r = −0.419, *p* = 0.02) were lost at multivariate analysis.

Baseline left ventricular GLS was associated with the number of NK cells (β = −0.362, *p* = 0.01). No significant correlates among immune cells were found for left ventricular GRS and global circumferential strain (GCS) at baseline. Independent correlates of the ΔGLS included the CD14++CD16+ monocytes (β = −0.423, *p* = 0.009) and CD4+ T-cells (β = 0.403, *p* = 0.01). The positive association at univariate analysis of the percentage of CD14++ monocytes with ΔGLS (*p* = 0.012, r = 0.455) and ΔGCS (*p* 0.04, r = 0.372) was subsequently lost at multivariate regression analysis. Independent correlates of left ventricular twist at baseline were total monocytes counts (β = −0.335, *p* = 0.04) and the percentage of CD14++CD16+ monocytes (β = 0.416, *p* = 0.01). Finally, only the percentage of the CD14++CD16+ monocytes was independently correlated to left ventricular untwist (β = −0.742, *p* = 0.09).

## 3. Discussion

There are substantial gaps in our understanding and addressing the unique mechanisms underlying the systemic impact of CKD on various organ systems both prior to and following organ transplantation [35,36]. Thus, identifications of novel, specific biological traits and clinical parameters which characterize CKD patients and KTRs would be of great significance and up to date. Accordingly the timing and the inceptive pathogenic immune mechanisms underlying the subclinical cardiovascular damage in CKD remain to be clarified. To the best of our knowledge, our exploratory study is the first to indicate independent correlations between cellular components of the innate and acquired immune system and specifically classical CD14++ monocytes, intermediate CD14++CD16+ monocytes, CD4+ T-cells, CD8+ T-cells and NK cells with the novel strain-related echocardiographic indices of subclinical myocardial dysfunction in CKD patients and KTRs without established CVD.

Monocyte subsets appear to be involved in the inflammatory pathways underlying the extracellular matrix deposition and cardiomyocyte hypertrophy as occur in diastolic dysfunction and HF with preserved EF. In vitro studies of human cardiac myofibroblasts co-cultured with peripheral blood monocytes isolated from healthy human donors, showed that direct cell–cell interaction between monocytes and cardiac myofibroblasts promotes TGF-β release and subsequently local matrix remodeling [37]. Taking into consideration models of hypertensive cardiomyopathy in order to draw potential similarities, a progressive decrease in the classical monocytes with a simultaneous increase in the percentage of CD16+ monocytes has been associated with increasing hypertension severity in hypertensive subjects [38]. Similarly, according to findings from the multiethnic study of atherosclerosis (MESA), increments in the classical CD14++CD16− monocytes are associated with declining of systolic blood pressure levels [39]. On the other hand, in our CKD patients, we found a direct association of RWT, a measure of left ventricular concentricity broadly used as an index of LVH, with CD14++CD16− monocytes count, whereas the correlation with arterial hypertension was lost at multivariate analysis. Similarly, an increased number of CD14++CD16− monocytes has been reported in patients with severe aortic valve stenosis, another model of concentric ventricular hypertrophy due to pressure overload [40]. Although these findings might appear contradictory at first sight, a plausible explanation would be the differential expression of monocyte subsets during the various phases of arterial hypertension pathogenesis and progression to target organ damage. Thus, a shift towards an increased expression of classical monocytes might occur during the development of left ventricular hypertrophy, which is a common denominator of arterial hypertension, aortic stenosis and uremic cardiomyopathy. Furthermore, we found that in KTR, the classical CD14++CD16− monocytes count was inversely associated with improvements in systolic wall motion indices, such as Sl and Sm as well as left ventricular EF following dipyridamole administration, albeit the significance of the latter was lost at multivariate analysis. Data from clinical studies are few and heterogenous in terms of the aims and the populations involved. Thus, in patients with acute myocardial infarction, peak levels of the classical CD14++CD16− monocytes have been inversely associated with the magnitude of myocardium salvaged as well as with the recovery of left ventricular function [41]. Of note, a decrease in classical monocytes counts has been observed following cardiac resynchronization therapy in the setting of HF, indicating a potential role of these cells in the myocardial remodeling process [42]. These findings along with ours underscore the relevance of CD14++CD16− monocytes in myocardial responses during various clinical settings. Nevertheless, further investigation is required to determine whether the classical monocytes play a direct role in myocardial injury and the adverse remodeling associated with kidney disease as well as clarify any potential singularities of the related inflammatory pathways in comparison to other disease states such as ischemic cardiomyopathy. An interesting finding of our study is the trend of independent correlations between the intermediate CD14++CD16+ monocytes with strain-related left ventricular myocardial performance indices, both in CKD patients and in KTRs. Thus, the CD14++CD16+ monocytes were independently associated with higher baseline GRS values in CKD patients, whereas in KTRs, they were associated with better baseline left ventricular twist and untwist parameters. Previous studies have shown elevated counts of the intermediate CD14++CD16+ monocytes in patients with both acute and stable chronic HF as well as direct associations of these cells with HF severity, hospitalizations and mortality [43]. Similarly, CD14++CD16+ monocytes are increased in patients with atrial fibrillations and are considered to promote fibrotic remodeling of the atria through increased expression of TGFβ [44,45]. However, studies aiming to shed light on the mechanisms of various monocyte subsets recruitment in the dysfunctional myocardium and their involvement in myocardial remodeling, hint to potentially protective features of the intermediate monocytes as well [46]. Notably, a less marked decrease in the CD14++CD16+ monocytes levels at the first month following acute myocardial infarction has been associated with a better left ventricular EF after six months [46]. Findings from a recent study also involving patients with acute myocardial infarction indicate a direct relationship between augmented collateral vessel formation and a higher percentage of CD14++CD16+ monocytes in the circulation, which subsequently translated into beneficial effects regarding infarct healing and left ventricular remodeling in these patients [47]. About our results, another potential explanation, though speculative, would be that a compensatory augmentation in strain-related myocardial performance indices is promoted in the setting of the microinflammatory milieu of CKD, which would subsequently lead to myocardial damage and remodeling. Our results are thought provoking and merit additional research considering the complex properties of the pro-inflammatory CD14++CD16+ monocytes in the pathogenesis of myocardial remodeling in CKD and following kidney transplantation. It is worth mentioning that among our patients, KTRs displayed a higher classical CD14++CD16− monocytes count together with a lower level of pro-inflammatory CD14++CD16+ monocytes compared to the CKD patients, which is in line with data from previous studies [48,49].

Among the T-cell subpopulations, it appears that the CD4+ T-cells are the dominant immune mediators involved in the remodeling process that occurs in the postischemic myocardium and in maladaptive myocardial hypertrophy via the release of fibrosis promoting cytokines such as IL-4 and IL-13 [26,50]. Nonetheless, there is paucity of clinical studies regarding the implications of T-cell subpopulations in CKD-related left ventricular remodeling. CD4+ T-cells producing interleukin-17 and interferon-γ are higher in hypertensive patients compared to healthy subjects [51,52]. About CKD, T-cell phenotyping in children with kidney dysfunction revealed an association between CD4+ T-cells senescence with improving diastolic function as assessed by E/E’ [30]. The results of our study pair well with the currently available evidence as presented above. Thus, we found a negative correlation of CD4+ T-cells with left ventricular EF in CKD patients as well as with the dipyridamole induced improvement of left ventricular EF in KTRs. Likewise, in KTRs we found a negative correlation of CD4+ T-cells both with MAPSE, a sensitive marker of early systolic dysfunction and TAPSE, a marker of right ventricular function, although the latter was lost following multivariate analysis. In accord with the above, with regard to strain-related indices of left ventricular function, CD4+ T-cells were inversely correlated with dipyridamole induced improvements in GLS in KTRs. Further characterization of the CD4+ T-cell responses is needed in order to discern possible pathogenic links to the development of uremic cardiomyopathy, evaluate their role as novel biomarkers of disease and subsequently examine the effects of immunomodulation on the CKD-related myocardial remodeling.

In contrast to CD4+ T-lymphocytes, the currently available evidence, albeit scarce, points to a complex role of the CD8+ T-cells in the development and progression of CVD. In experimental arterial hypertension models, wild type mice undergoing adoptive transfer of CD8+ T-cells from hypertensive mice developed salt sensitive hypertension [13,53,54]. Specifically, IFNγ is considered to largely mediate the interaction of CD8+ T-cells with the nephron structures, eventually leading to sodium retention [53,54]. Furthermore, an increased proportion of immunosenescent, proinflammatory CD8+ T-cells have been found in hypertensive patients [55]. With regard to myocardial remodeling, post-myocardial infarction mice lacking functional CD8^+^ T-cells displayed delayed removal of necrotic debris and defective scar formation, inferring a profibrotic role of CD8+ T-cells in the myocardium [56]. In contrast, in mice submitted to transverse aortic constriction, the absence of CD8+ T-cells but not of CD4+ T, cells did not prevent either excessive collagen deposition in the myocardium or left ventricular chamber dilation and systolic dysfunction [50]. We found an independent association of CD8+ T-cells with both left ventricular twist and untwist in patients with CKD. Notably, left ventricular torsion as represented by the systolic twist and the diastolic untwist rates, is greater in hypertensive than normotensive individuals, which might be ascribed to a compensatory mechanism in the setting of increased aortic stiffness during the early stages of hypertensive cardiomyopathy [6]. Furthermore, augmented left ventricular twist and twist rates have been observed in CKD patients and appear to be compensatory to impairments in GLS and GRS [57]. Considering the differences in the pathogenic models implemented by the above studies as well as the preliminary nature of our results, it remains to be determined whether CD8+ T-cells are simple bystanders or active and independent players in the mechanisms of CKD-related myocardial dysfunction.

Studies on NK cells in HF are very limited as well. Interestingly, we detected an independent correlation of NK cells both with E/A ratio as well as with GLS in KTRs only. These results appear controversial at first sight considering that a physiological reduction in E/A ratio is observed with aging, whereas the relationship of this index with left ventricular diastolic function is more complex and should be evaluated in combination with other markers, including the E/E’ ratio. Accordingly, in a previous study of patients undergoing peritoneal dialysis, we found that patients with higher NK cell levels had a higher E/E’ ratio [58]. Similarly, results from the multiethnic study of atherosclerosis point out a direct relationship between increments in NK cells with higher average systolic blood pressure levels [37]. On the other hand, we also found that higher NK cell counts were associated with improved left ventricular strain in KTRs. Experimental data point out a cardioprotective, antifibrotic role of NK cells in the setting of HF, via production of IFNγ, suppression of cardiac myocyte apoptosis and collagen deposition, as well as increases in neovascularization [59,60]. Although depletion together with modulation of the phenotypic and cytotoxic characteristics of NK cells may contribute to the immune dysfunction in advanced CKD, the role of this alterations in the pathogenesis of CKD associated CVD remains currently hypothetical and requires further investigation [61,62].

Our study provides preliminary but noteworthy evidence regarding the potential associations between a selected panel of immune cell subsets in the circulation with an array of echocardiographic indices of subclinical myocardial dysfunction in CKD patients and KTRs. The main strength of our study is its design in terms of including a cohort of two homogenous patient groups, CKD patients and KTRs, respectively, in terms of clinical characteristics and specifically with no clinically established CVD. Nonetheless, there are some limitations that need to be mentioned. First, the sample size was relatively small and recruited from one single center. Second, the observational and cross-sectional nature of this study precludes us from drawing conclusions about causality in the associations detected between immune cell subsets and conventional and novel deformation-related indices of left ventricular function in our patients. Taking into consideration the associations we found between immune cell populations and markers of cardiomyopathy, we intend to prospectively investigate these findings in our patients. Overall, studies of longitudinal design and with larger sample sizes are needed, so as to further clarify a potential causal relationship between alterations in the expression of immune cell subsets and progression of CKD-related myocardial dysfunction. Moreover, we examined the expression of a selected panel of immune cell subsets in the peripheral blood; however, the alterations of their functional characteristics and respective potential consequences remain to be assessed. Thus, future investigation should incorporate functional analyses in order to elucidate the activity and functional properties of immune cell subsets and to reveal their specific roles in myocardial pathology.

## 4. Materials and Methods

### 4.1. Study Cohort

**Figure 3:**
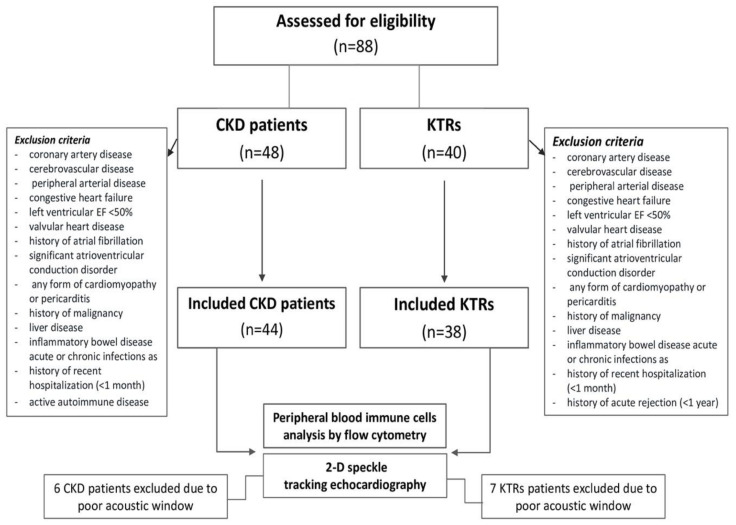
Flowchart of this study.

We conducted a single-center observational cohort study on 44 consecutive patients with CKD and without established cardiovascular disease who were under regular follow-up by our outpatient nephrology clinic and 38 kidney transplant recipients (KTRs) without established cardiovascular disease under follow-up by the kidney transplant unit of our hospital (Figure 3). Definition of established cardiovascular disease as an exclusion criterion included history of atherosclerotic cardiovascular disease such as coronary artery disease, cerebrovascular as well as peripheral arterial disease, history of congestive HF or reduced left ventricular EF < 50%, the presence of moderate-severe valvular heart disease, history of atrial fibrillation or significant atrioventricular conduction disorder, as well as any form of cardiomyopathy or pericarditis. Furthermore, patients with a history of malignancy, liver disease, inflammatory bowel disease, acute or chronic infections as well as a history of recent hospitalization (<1 month) were excluded from this study. Specifically, regarding CKD patients, additional exclusion criteria included active autoimmune disease requiring current treatment with steroids and/or other immunosuppressive medications. With regard to KTRs, those with a history of acute rejection (<1 year) were further excluded from this study. The Ethical Committee of the University Hospital of Ioannina approved the study protocol (5/26-3-2020) and all participants provided fully informed consent.

### 4.2. Evaluation of Immune Cell Subpopulations by Flow Cytometry

**Figure 4:**
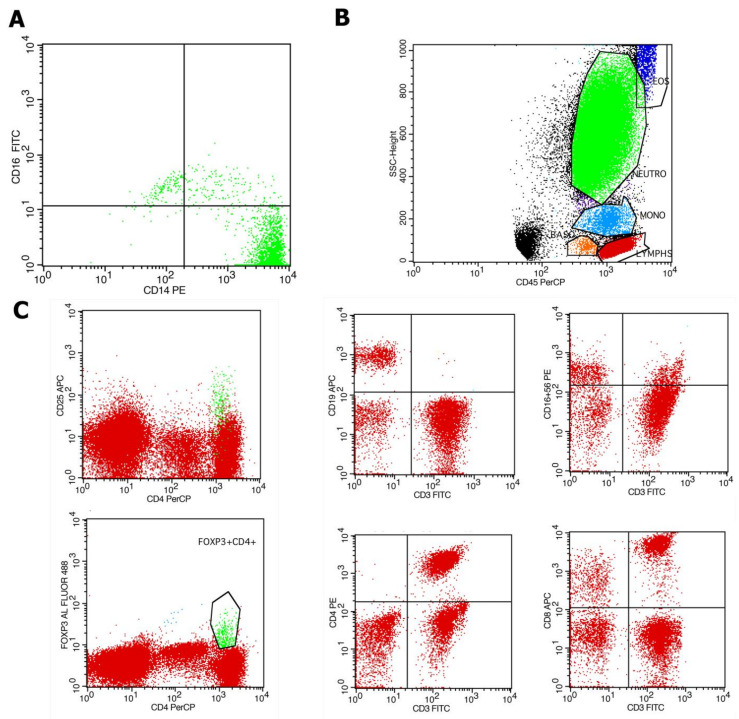
Flow cytometric analysis of a patient with CKD. (**A**) Representative dot plots depicting monocyte subsets (green color) according to surface expression of CD14 and CD16 in CD14++CD16−, CD14++C16+, and CD14+CD16++ subpopulations. (**B**) Representative dot plots depicting lymphocyte gating (red color) with B-lymphocytes, T-lymphocytes, and natural killer (NK) cells defined as CD16+CD56+ cells, CD4+ T-cells, and CD8+ T-cells. (**C**). Representative dot plots depicting T regulatory cells (Tregs) defined as CD4+ FoxP3+ CD25high positive cells (green color).

The analysis of peripheral blood immune cells was conducted by flow cytometry (FC) in a 100 μL whole-blood assay using 100 μL of whole blood and within 8 h from blood specimen withdrawal. Ethylenediaminetetraacetic acid (EDTA) blood-collecting tubes were utilized for the collection of 2 mL of whole-blood samples from patients under standardized conditions. The following conjugated monoclonal antibodies were used for analysis: CD45(BD), CD14(BD), CD16(BD), CD4(BD), CD8(BD), CD56(BD), CD3(BD), CD19(BD), CD25(BD), and Fox-P3(eBioscienceTM). Immune cell subpopulations were analyzed by standard techniques with flow cytometry FC (FACSCalibur) and Cell Quest and FACSDiva software V9.0 (BD Biosciences, Franklin Lakes, NJ, USA). 100 μL of whole blood was added to flow cytometry (FC) tubes and incubated with respective antibodies according to the manufacturer’s instructions. Subsequently, 500 μL of Versalyse (Beckman Coulter, Brea, CA, United States) was added and incubated for 10 min at room temperature (18–25 °C) protected from light, to lyse red blood cells. Samples were processed immediately for flow cytometry analysis. The data were analyzed using the CellQuest V3.1 software (Becton Dickinson, Franklin Lakes, NJ, USA). Accordingly, the percentage and the absolute number of CD14++CD16− monocytes, CD14++CD16+ monocytes and CD14+16++ monocyte subsets out of the total monocytes were measured. Similarly, the percentage and absolute number of NK cells (CD3+CD16+56+), CD3-CD19+ B lymphocytes, CD3+ CD4+ T-cells, CD3+CD8+ T-cells, and Tregs (CD4+CD25+ FoxP3+) out of total lymphocytes were measured (Figure 4).

### 4.3. Echocardiographic Evaluation

The echocardiographic evaluation was performed by a single operator using a Vivid 7 ultrasound machine (GE Vingmed ultrasound AS) in all patients. Standard echocardiographic views were used and acquired images and video loops were stored digitally in high analysis. A single observer blinded of the patients’ identity performed offline analysis using EchoPac (version 113—GE Vingmed ultrasound AS). The echocardiographic studies were performed within 1 month from immune cell subset analysis. Initially, a basic echocardiogram was performed, and classical systolic and diastolic indices of ventricular function were obtained according to the European Society of Cardiology and European Association of Cardiovascular Imaging guidelines [63]. In addition, a two-dimensional STE analysis was performed in both parasternal and apical views (at frame rates 60–90 Hz). The endocardial left ventricular borders were manually traced (region of interest). Two-dimensional STE analysis included assessment of global longitudinal strain, as well as global radial and circumferential strain. Left ventricular twist was calculated as the difference between apical and basal left ventricular rotation as it was assessed from equivalent short-axis views. Untwist rate was measured as the peak negative time derivative of twist during diastole. Following the baseline echocardiographic evaluation, infusion of dipyridamole for 6 min (0.84 mg/kg) was performed. A new echocardiographic assessment (focused mainly on left ventricular systolic and diastolic function indices) was performed [64]. At the end of the dipyridamole infusion, 125–250 mg of aminophylline was administered to the patient, to counteract any dipyridamole negative effect. Finally, the differences (Δ) between the values of measured echocardiographic parameters post and prior to dipyridamole infusion were calculated.

### 4.4. Clinical and Laboratory Assessment

Anthropometric and clinical data were recorded at baseline by patients’ medical records, including comorbidities such as the presence of diabetes mellitus (DM), arterial hypertension and medications. Specifically, regarding KTRs, transplantation and dialysis vintage as well as the immunosuppressive regimen were recorded. Simultaneously with flow cytometry analysis, common biochemical parameters were measured in accordance with standard methods applied in the hospital laboratory. Complete blood counts and classical inflammatory markers including C-reactive protein (CRP) and erythrocyte sedimentation rate (ESR), were measured. Urinary protein to creatinine ratio (UPCR) measurement assessment was performed in morning spot urine samples. Whole-blood levels of calcineurin inhibitors (CNIs)—cyclosporine and tacrolimus)—were measured in KTRs using high-performance liquid chromatography (HPLC).

### 4.5. Statistical Analysis

Continuous parameters are presented as the mean ± standard deviation in normally distributed variables or median (Interquartile range—IQR) in variables without normal distribution. Dichotomous parameters are presented as frequency (percentage). Normal distribution of all continuous variables was tested with the parametric Shapiro–Wilk normality test. Differences between groups were determined by the independent-samples t test or the non-parametric Mann–Whitney test in continuous variables and the chi-square followed by Fisher’s exact test for categorical variables (frequency distributions). Univariate association analysis was performed by assessing the Pearson or Spearman correlation coefficients, as indicated. Linear regression model analysis was used to adjust for confounders in cell subtype differences among patient subgroups. Multivariate association analysis was performed using stepwise linear regression analysis models that included all variables with a univariate association at the level of *p* value < 0.1. *p* values were always two sided and a value of *p* < 0.05 was considered significant. The SPSS statistical software package (IBM SPSS Statistics, Version 23) was used.

## 5. Conclusions

Immune cell subsets, including classical CD14++CD16 and intermediate CD14++CD16+ monocytes, NK cells and lymphocytes subsets independently correlate with subclinical indices of myocardial dysfunction, including left ventricular strain and torsion-related parameters, in patients with CKD and in KTRs without established CVD. Our findings provide novel insights suggesting a potential role for specific immune cell phenotypes for CKD-related cardiomyopathy. Taking into consideration that subtle alterations in left ventricular function commence early in CKD, future mechanistic studies are required to shed light on the potential pathophysiological significance of the role of specific immune cell subsets during the initial phases of myocardial remodeling in these patients. Prospective studies are highly needed to clarify the utility of immune cell populations as potential prognostic markers for development of cardiomyopathy. Finally, development of interventions that modulate the expression and activity of specific immune cell subsets might represent a new target of remarkable therapeutic prospect for uremic cardiomyopathy.

## Figures and Tables

**Table 1:** Main clinical characteristics in all patients, in kidney transplant recipients (KTRs) and in CKD patients. Statistically significant differences between subgroups are highlighted in bold.

	All Patients (N = 82)	CKD Patients (N = 44)	KTRs (N = 38)	*p*-Value *
Age (years)	58 ± 11	63 ± 11	53 ± 9	<0.001
Males, N (%)		28 (64)	27 (71)	
DM, N (%)	26 (32)	17 (39)	9 (24)	0.147
Arterial Hypertension, N (%)	71 (86)	39 (88)	32 (84)	0.558
Transplantation vintage	-	-	77.5 (58–111)	-
eGFR (mL/min/1.73 m^2^)	42 (20–57)	24 (15–41)	55 (48–72)	**<0.001**
UPCR (g protein/g creatinine)	0.32 (0.13–1.92)	1.31 (0.23–2.61)	0.16 (0.09–0.56)	**<0.001**
Hemoglobin (g/dL)	12.9 ± 2.0	12.4 ± 1.9	13.4 ± 2.0	**0.028**
Uric acid (mg/dL)	7.1 ± 1.7	7.4 ± 1.8	6.8 ± 1.5	0.123
ESR (mm/hour)	24 (13–34)	30 (21–42)	15 (12–26)	**0.001**
CRP (mg/L)	3 (2–6)	3 (2–6)	3.5 (3–7)	0.442
Glucose (mg/dL)	101 (91–115)	100 (89–114)	101 (93–118)	0.429
Albumin (g/dL)	4.2 (4–4.5)	4.2 (3.8–4.4)	4.2 (4–4.5)	0.253
Total proteins (g/dL)	7.0 ± 0.6	7.1 ± 0.6	7.0 ± 0.5	0.614
Total cholesterol (mg/dL)	187 ± 37	183 ± 43	190 ± 28	0.339
Triglycerides (mg/dL)	144 (113–192)	150 (113–200)	142 (113–166)	0.468
LDL cholesterol (mg/dL)	109 ± 35	108 ± 41	109 ± 26	0.835
HDL cholesterol (mg/dL)	46 (40–55)	43 (36–50)	50 (44–62)	**0.001**
Ferritin (ng/mL)	65 (40–108)	79 (48–112)	57 (31–104)	0.200
Calcium (mg/dL)	9.5 (9.1–9.7)	9.3 (8.8–9.6)	9.7 (9.4–10.1)	**<0.001**
Phosphorus (mg/dL)	3.4 (2.8–4.1)	3.9 (3.3–4.6)	2.9 (2.7–3.5)	**<0.001**
iPTH (pg/mL)	121 (85–226)	156 (88–294)	109 (74–169)	**0.048**
Cyclosporine N (%)	-	-	15 (40)	-
Tacrolimus N (%)	-	-	23 (60)	-
Statins N (%)	57 (70)	29 (66)	28 (74)	0.464
ACEI/ARB N (%)	48 (58)	25 (57)	23 (60)	0.656
B-blockers N (%)	49 (60)	23 (52)	26 (68)	0.115

Values are expressed as the mean (±SD) or median (IQR 25–75th percentiles). ACEI/ARB, angiotensin-converting enzyme inhibitors/angiotensin receptor blockers; CKD, chronic kidney disease; CRP, C-reactive protein; eGFR, estimated glomerular filtration rate; ESR, erythrocyte sedimentation rate; iPTH, intact parathyroid hormone; N, number; UPCR, urinary protein to creatinine ratio. * *p* refers to *t* test significance for normal distribution variables, to the Mann–Whitney test significance for non-parametric variables, or to chi square test significance for categorical variables.

**Table 2:** Immune cell subpopulations in all patients, in kidney transplant recipients (KTRs) and in CKD patients. Statistically significant differences between subgroups are highlighted in bold.

	All Patients (N = 82)	CKD Patients (N = 44)	KTRs (N = 38)	*p*-Value *
WBC (N)	7485 (6080–9260)	7045 (5745–8925)	7710 (6920–10790)	**0.023**
Monocytes (N)	500 (400–600)	400 (300–600)	600 (400–800)	**0.001**
Monocytes (%)	6.7 (5.4–7.9)	6.4 (5.3–7.4)	7.1 (5.9–8.7)	**0.033**
CD14++CD16− (N)	415 (318–522)	366 (258–438)	479 (354–599)	**0.001**
CD14++CD16− (%)	83.7 (79.2–88.2)	81.7 (75.9–85.6)	87.1 (83.6–90.1)	**<0.001**
CD14++CD16+ (N)	31 (18–48)	35 (24–53)	25 (16–45)	0.095
CD14++CD16+ (%)	6.5 (3.6–9.2)	8.2 (5.9–11.3)	4.6 (2.8–7.3)	**<0.001**
CD14+CD16++ (N)	22 (15–32)	25 (19–36)	18 (13–28)	**0.012**
CD14+CD16++ (%)	4.6 (3.1–6.9)	5.8 (4.3–8.2)	3.2 (1.9–5.4)	**<0.001**
Lymphocytes (N)	1845 (1520–2560)	1790 (1585–2405)	2000 (1450–2710)	0.451
Lymphocytes (%)	25.6 ± 8.0	26.4 ± 7.5	24.7 ± 8.5	0.335
T-lymphocytes (N)	1474 (1125–2053)	1376 (1114–1796)	1732 (1156–2228)	0.173
T-lymphocytes (%)	79.0 ± 9.2	76.7 ± 9.6	81.4 ± 8.3	**0.026**
B-lymphocytes (N)	91 (48–157)	94 (61–161)	88 (33–140)	0.196
B-lymphocytes (%)	4.8 (3.2–7.4)	5.6 (3.7–7.9)	4.3 1.9–6.7)	**0.042**
NK cells (N)	276 (170–358)	304 (178–370)	257 (150–324)	0.201
NK cells (%)	14.4 (9.7–18.1)	16.5 (11.3–19.3)	13.2 (7.9–18.8)	0.056
CD4+ T-cells (N)	830 (608–1187)	830 (595–1101)	835 (610–1299)	0.395
CD4+ T-cells (%)	46.2 ± 9.9	45.2 ± 10.2	47.4 ± 9.6	0.321
CD8+ T-cells (N)	582 (447–838)	567 (411–781)	612 (448–896)	0.370
CD8+ T-cells (%)	32.7 (26.5–37.1)	32.1 (25.0–37.3)	33.1 (28.4–37.1)	0.491
Tregs (N)	23 (15–39)	33 (19–48)	19 (13–28)	**0.002**
T Regs (%)	1.47 (0.81–2.02)	1.75 (1.13–2.44)	0.93 (0.63–1.71)	**<0.001**

Values are expressed as the mean (±SD) or median (IQR 25–75th percentiles). CD, cluster of differentiation; NK, natural killer; N, number per μL; Tregs, T regulatory cells. * *p* refers to t test significance for normal distribution variables, to the Mann–Whitney test significance for non-parametric variables, or to chi square test significance for categorical variables.

**Table 3:** Univariate and multivariate association analysis of classical and novel echocardiographic indices and their changes post-dipyridamole with immune cell subsets in CKD patients (only variables with significant associations are shown).

Univariate Analysis			Multivariate Analysis		
	R/Rho	*p* Value	β	*p* Value	ANOVA R^2^ *p* Value
**RWT**					0.338, *p* = 0.001
*CD14++CD16− monocytes (N)*	*0.638*	*0.000*	*0.447*	***0.004***	
*B lymphocytes (%)*	*−0.363*	*0.030*	*−0.328*	***0.03***	
WBC	0.41	0.013	−0.92	0.67	
Monocytes (N)	0.559	0.000	−0.02	0.997	
CD14+CD16++ monocytes (%)	−0.53	0.001	−0.240	0.181	
Lymphocytes (%)	−0.317	0.060	−0.151	0.309	
Arterial hypertension	0.484	0.003	0.241	0.109	
Albumin	−0.441	0.007	−0.176	0.298	
Proteins	−0.383	0.023	−0.174	0.259	
HDL	−0.356	0.033	−0.176	0.258	
Calcium	−0.338	0.044	*−0.150*	*0.320*	
**LVEF**					0.186, *p* = 0.009
*CD4+ T-cells (N)*	*−0.451*	*0.006*	*−0.431*	***0.009***	
T-cells No	−0.364	0.029	0.012	0.969	
Lymphocytes (%)	−0.353	0.035	−0.171	0.377	
Lymphocytes (N)	−0.345	0.04			
Hemoglobin	−0.317	0.059	−0.191	0.260	
** ΔMAPSE septal **					0.360, *p* = 0.001
*CD14++CD16+ monocytes (N)*	*−0.468*	*0.004*	*−0.405*	***0.007***	
Urea	−0.333	0.048	−0.263	0.074	
UPCR	−0.399	0.016	−0.155	0.333	
Arterial hypertension	−0.363	0.030	−0.276	0.070	
*HDL*	*0.340*	*0.043*	*0.413*	***0.006***	
**GRS**					
*CD14++CD16+ monocytes (%)*	*0.351*	*0.041*			
*CD14++CD16+ monocytes (N)*	*0.042*	*0.015*			
**TWIST**					0.164, *p* = 0.02
*CD8+ T-cells (%)*	*0.309*	*0.080*	*0.405*	***0.021***	
Glucose	0.398	0.028	−0.191	0.261	
Triglycerides	−0.348	0.051	−0.278	0.098	
**UNTWIST**					0.135, *p* = 0.03
*CD8+ T-cells (%)*	*−0.371*	*0.033*	−0.367	**0.036**	
Age	−0.389	0.025	−0.287	0.086	

GRS, global radial strain; KTRs, kidney transplant recipients, LDL, low-density lipoprotein; LVEF, left ventricle ejection fraction; MAPSE, mitral annular plane systolic excursion; N, number; RWT, relative wall thickness; UPCR, urine protein to creatinine ratio.

**Table 4:** Univariate and multivariate association analysis of classical and novel echocardiographic indices and their changes post-dipyridamole with immune cell subsets in kidney transplant recipients (only variables with significant associations are shown).

Univariate Correlations			Multivariate Correlations		
	R/Rho	*p*	Multivariate β	*p*	ANOVA R^2^ *p* Value
**LVEF**					0.357, *p* = 0.002
*Tregs (%)*	*−0.384*	*0.033*	*−0.341*	***0.033***	
*Female gender*	*0.456*	*0.01*	*0.454*	***0.006***	
CD14++CD16+ monocytes (N)	−0.351	0.05	−0.150	0.350	
**ΔLVEF**					0.319, *p* = 0.005
*Albumin*	*0.447*	*0.012*	*0.353*	***0.034***	
LDL	0.381	0.034	0.354	0.06	
CD14++CD16− (%)	−0.447	0.036	−0.174	0.179	
T-cells (%)	−0.475	0.007	0.95	0.557	
*CD4+ T-cells (N)*	*−0.483*	*0.006*	***−0.378***	***0.024***	
CD8+ T-cells (N)	−0.371	0.004	−0.262	0.140	
**TAPSE**					0.288, *p* = 0.001
*CD8+ T-cells (%)*	*0.494*	*0.005*	*0.559*	***0.001***	
CD4+ T-cells (N)	−0.456	0.001	−0.043	0.805	
Glucose	0.404	0.024	0.221	0.124	
ESR	0.349	0.050	0.239	0.161	
**MAPSE septal**					0.483, *p* = 0.000
Arterial Hypertension	−0.364	0.044	−0.13	0.414	
*HDL*	*0.417*	*0.017*	*0.474*	***0.002***	
*Phosphorus*	*0.369*	*0.041*	*0.376*	***0.013***	
Albumin	0.331	0.069	0.87	0.582	
*CD4+ T-cells (%)*	*−0.303*	*0.098*	*−0.359*	***0.016***	
**ΔSm**					0.266, *p* = 0.01
CD14+CD16++ monocytes (N)	0.643	0.010	0.151	0.576	
*CD14++ monocytes (%)*	*−0.489*	*0.010*	*−0.516*	***0.010***	
**ΔSl**					0.500, *p* = 0.000
Age	0.439	0.032	0.137	0.405	
*CD14++CD16− monocytes (%)*	*−0.747*	*0.000*	*−0.707*	***0.000***	
C14++CD16+ monocytes (%)	0.484	0.017	0.104	0.519	
C14+CD16++ monocytes %	0.466	0.029	−0.068	0.762	
Calcium	0.451	0.027	0.200	0.217	
**E/A**					0.350, *p* = 0.02
*Age*	*−0.466*	*0.008*	*−0.481*	***0.004***	
Transplant vintage	0.596	0.000	0.211	0.179	
*NK cells (N)*	*−0.359*	*0.047*	*−0.387*	***0.017***	
Hemoglobin	−0.429	0.016	−0.140	0.435	
**GLS**					0.271, *p* = 0.01
*Gender male*	*0.443*	*0.017*	*0.434*	***0.015***	
*NK cells (N)*	*−0.447*	*0.013*	*−0.362*	***0.038***	
LDL	0.384	0.036	0.180	0.311	
**ΔGLS**					0.435, *p* = 0.002
* Hypertension *	*0.315*	*0.09*	0.364	**0.022**	
CD14++CD16− monocytes (%)	0.455	0.012	0.248	0.156	
*CD14++CD16+ monocytes (%)*	*- 0.374*	*0.042*	*−0.423*	***0.009***	
*CD4+ T-cells (%)*	0.386	0.035	*0.403*	***0.012***	
CD4+ T-cells (N)	0.386	0.04			
Tregs (%)	0.324	0.081	0.274	0.107	
Albumin	−0.320	0.085	−0.260	0.097	
**TWIST**					0.345, *p* = 0.003
*Monocytes (N)*	*−0.412*	*0.024*	*−0.335*	***0.045***	
*CD14++CD16+ monocytes (%)*	*0.442*	*0.015*	*0.416*	***0.015***	
Lymphocytes No	−0.343	0.064	−0.03	0.874	
**UNTWIST**					0.550, *p* = 0.009
DM	*0.326*	*0.079*	0.113	0.647	
*CD14++CD16+ monocytes (%)*	*−0.400*	*0.029*	*−0.742*	***0.009***	
Ferritin	−0.344	0.063	−0.360	0.116	
Cyclosporine C0	−0.545	0.083	−0.198	0.436	

C0, trough blood levels; DM, diabetes mellitus; E/A, early to late diastolic transmitral wave ratio; GCS, global circumferential strain; GLS, global longitudinal strain; LDL, low-density lipoprotein; LVEF, left ventricle ejection fraction; MAPSE, mitral annular plane systolic excursion; No, number; Sm, medial wall systolic velocity; Sl, lateral wall systolic velocity; TAPSE, tricuspid annular plane systolic excursion; Δ, difference between values of echocardiographic parameters post and prior to dipyridamole infusion.

## Data Availability

Main relevant data are contained within this article. Additional data regarding methods and results presented in this study are available on request from the corresponding author.

## Appendix A

**Table A1:** Associations of immune cell subpopulations in CKD patients with markers of kidney function, inflammatory markers and indices of CKD-MBD.

CD14++CD16+ (N)	NK Cells (N)	T-Lymphocytes (N)
Phosphorus rho = 0.436, *p = 0.003*	UPCR rho = −0.302, *p = 0.04* Hemoglobin rho = 0.319, *p = 0.03* Ferritin rho = 0.307, *p = 0.04* Calcium rho = 0.329, *p = 0.03* iPTH rho = −0.327, *p = 0.03*	eGFR rho = 0.328, *p = 0.03* Creatinine rho = −0.361, *p = 0.02* Hemoglobin rho = 0.437, *p = 0.003* CRP rho = −0.344, *p = 0.02*
**CD14++CD16+ (%)**	**Lymphocytes (N)**	**CD4+ T-Cells (N)**
Phosphorus rho = 0.410, *p = 0.006*	eGFR rho = 0.388, *p = 0.009* Urea rho = −0.374, *p = 0.01* Creatinine rho = −0.408, *p = 0.006* Hemoglobin rho = 0.469, *p = 0.001* Calcium rho = 0.378, *p = 0.01*	eGFR rho = 0.495, *p* = 0.001 Urea rho = −0.381, *p* = 0.01, Creatinine rho = −0.505, *p* <0.001 Hemoglobin rho = 0.522, *p* < 0.001 Calcium rho = 0.371, *p* = 0.01 PTH rho = −0.324, *p* = 0.03
**CD14+CD16++ (N)**	**Lymphocytes (%)**	**CD8+ T Cells (N)**
Urea rho = 0.298, *p = 0.005*	UPCR rho = −0.439, *p = 0.003* eGFR rho = 0.395, *p = 0.008* Urea rho = −0.408, *p = 0.006* Creatinine rho = −0.475, *p = 0.001* Hemoglobin rho = 0.370, *p* = *0.013* Calcium rho = 0.478, *p = 0.008*	Urea rho = −0.345, *p = 0.02* Ferritin rho = −0.318, *p = 0.04*

Only correlations reaching statistical significance are presented. eGFR, estimated glomerular filtration rate; iPTH, intact parathyroid hormone; UPCR, urine protein to creatinine ratio.

**Table A2:** Associations of immune cell subpopulations in kidney transplant recipients with markers of kidney function, inflammatory markers, indices of CKD-MBD and blood levels of calcineurin inhibitors.

CD14++%	NK Cells (%)	CD4+ T-Cells (%)
Hemoglobin rho = −0.37, *p = 0.05* Glucose rho = −0.33, *p = 0.04* Calcium rho = −0.39, *p = 0.014*	ESR rho = 0.393, *p = 0.01*	Tacrolimus C0 rho = 0.708, *p* < 0.001
**CD14++CD16− (N)**	**Lymphocytes (N)**	**CD8+ T Cells (N)**
iPTH rho = −0.328, *p = 0.04*	eGFR rho = 0.359 *p = 0.02* Cyclosporine C0 rho = −0.594, *p = 0.02*	Hemoglobin rho = 0.358, *p = 0.02* eGFR rho = 0.362, *p = 0.02*
**CD14++CD16+ (%)**	**Lymphocytes (%)**	**B-Lymphocytes (N)**
Phosphorus rho = 0.33, *p = 0.04*	eGFR rho = 0.359 *p = 0.02* Cyclosporine C0 rho = −0.594, *p = 0.02*	Phosphorus rho = −0.430, *p = 0.007* Urea rho = −0.426, *p = 0.008* Creatinine rho = −0.369, *p = 0.02* UPCR rho = −0.335, *p = 0.039* Cyclosporine C0 rho = 0.686, *p = 0.007*
**CD14+CD16++ (N)**	**T-Lymphocytes (N)**	**B-Lymphocytes (%)**
Calcium rho = 0.388, *p =0.01*	eGFR rho = 0.376 *p = 0.02* Cyclosporine C0 Rho = −0.550, *p = 0.04* Tacrolimus C0 rho = 0.439, *p = 0.03*	Phosphorus rho = −0.411, *p = 0.01* Urea rho = −0.349, *p = 0.03* UPCR rho = −0.405, *p = 0.01*
**CD14+CD16++ (%)**	**T-Lymphocytes (%)**	**Tregs (%)**
Calcium rho = 0.388, *p = 0.01*	Cyclosporine C2 rho = 0.609, *p = 0.02* Tacrolimus C0 rho = 0.419, *p = 0.047*	Tacrolimus C0 rho = 0.504, *p = 0.01*
**NK Cells (N)**	**CD4+ T Cells (N)**	
ESR rho = 0.395, *p = 0.01* Calcium rho = 0.356, *p = 0.028*	iPTH (N) rho = −0.340, *p = 0.03*	

Only correlations reaching statistical significance are presented. C0, trough blood levels; C2, blood levels 2 h post-dose; eGFR, estimated glomerular filtration rate; ESR, erythrocyte sedimentation rate; iPTH, intact parathyroid hormone; UPCR, urine protein to creatinine ratio.

**Table A3:** Echocardiographic parameters at baseline and differences between respective values following and prior to dipyridamole infusion (Δ) in CKD patients and kidney transplant recipients.

	CKD Patients		KTRs	
	**Baseline**	**Δ**	**Baseline**	**Δ**
LAVI, mL/m^2^	32.6 ± 10.2	NA	32.2 ± 10.6	NA
LVMI, g/m^2^	112.3 ± 36.5	NA	99.09 (84.3–134.6)	NA
RWT	0.46 (0.42–0.54)	NA	0.46 (0.38–0.51)	NA
EF, %	68 ± 7	3 ± 9	65 ± 7	10 ± 7
TAPSE, cm	2.5 ± 0.43	0.05 ± 0.44	2.2 (1.9–2.4)	0.14 ±0.46
MAPSE septal, cm	1.3 (1.2–1.6)	−0.26 ± 0.35	1.32 ± 0.29	0.14 ± 0.24
MAPSE lateral, cm	1.7 ± 0.3	0.43 ± 0.32	1.6 ± 0.3	0.029 ± 0.27
E/A	0.80 (0.65–97)	−0.05 (−0.13–0.09)	0.91 ±0.24	−0.05 (−0.19–0.16)
E’ average, cm	0.09 ± 0.01	0.012 ± 0.020	0.088 ± 0.018	0.023 (0.010–0.040)
E/E’	8.4 ± 2.8	0.1 (−1.3–1.5)	8.8 (7.6–9.6)	−1.2 (−2.7–0.7)
Sm, cm/s	0.08 (0.06–0.09)	0.01 ± 0.02	0.08 (0.07–0.09)	0.02 (0–0.04)
Sl, cm/s	0.09 (0.08–0.11)	0.02 (0.00–0.02)	0.09 (0.08–0.1)	0.017 (±0.024)
GLS, %	−20.3 ± 3.1	−1.9 ± 4.1	−21.1 (−21.9–−18.1)	−2.5 ± 3.3
GRS, %	27.9 ±15.3	0.0 (−11.0–15.7)	21.9 (13.2–37.4)	−1.84 ±21.4
GCS, %	−25.9 (−30.6–−21.0)	−3.0 ± 7.8	−28.7 ± 7.0	−1.73 ± 9.2
TWIST, degrees	9.1 ± 4.3	−1.1 (−2.7–3.2)	6.2 (3.4–9.5)	3.2 ± 6.7
UNTWIST, degrees/s	−77.6 ± 34.1	−10.5 ± 40.2	−55.0 (−88.9–−34.4)	−51.4 ± 48

Values are expressed as the mean (±SD) or median (IQR 25–75th percentiles). CFR, coronary flow reserve; E’, early diastolic tissue wave velocity; E/A, early to late diastolic transmitral wave ratio; GCS, global circumferential strain; GLS, global longitudinal strain; GRS, global radial strain; kidney transplant recipients, KTRs, kidney transplant recipients, LAVI, left atrial volume index; LVEF, left ventricle ejection fraction; LVMI, left ventricle mass index; MAPSE, mitral annular plane systolic excursion; NA, non available; RWT, relative wall thickness; Sm, medial wall systolic velocity; Sl, lateral wall systolic velocity; TAPSE, tricuspid annular plane systolic excursion; Δ, difference between values of echocardiographic parameters post and prior to dipyridamole infusion.
